# Comprehensive analysis of the expression level, prognostic value, and immune infiltration of cuproptosis-related genes in human breast cancer

**DOI:** 10.1097/MD.0000000000040132

**Published:** 2024-10-18

**Authors:** Jian Chen, Wei Cao, Yingliang Li, Jia Zhu

**Affiliations:** a Breast Disease Center, The First Affiliated Hospital, Jiangxi Medical College, Nanchang University, Nanchang, China; b Department of Emergency Medicine, The First Affiliated Hospital of USTC, Division of Life Sciences and Medicine, University of Science and Technology of China, Hefei, China.

**Keywords:** BRCA, breast cancer, copper, cuproptosis, immune cell infiltration, prognosis

## Abstract

**Background:**

As a novel cell death form, cuproptosis results from copper combining with lipidated proteins in the tricarboxylic acid cycle. To the best of our knowledge no study has yet comprehensively analyzed the relationship between cuproptosis-related genes and breast cancer.

**Methods:**

The expression, prognostic value, mutations, chemosensitivity, and immune infiltration of cuproptosis-related genes in breast carcinoma patients were analyzed, PPI networks were constructed, and enrichment analyses were performed based on these genes. TIMER, UALCAN, Kaplan–Meier plotter, Human Protein Atlas, cBioPortal, STRING, GeneMANIA, DAVID, and R program v4.0.3 were used to accomplish the analyses above.

**Results:**

Compared to normal breast tissues, FDX1, LIAS, LIPT1, DLD, DLAT, PDHA1, MTF1, and GLS were down-regulated in breast cancer tissues, while CDKN2A was up-regulated. High expression of FDX1, LIAS, DLD, DLAT, MTF1, GLS, and CDKN2A were associated with favorable overall survival. Cuproptosis-related genes showed a high alteration rate (51.3%) in breast cancer, contributing to worse clinical outcomes. The expression levels of FDX1, LIPT1, DLD, DLAT, PDHA1, PDHB, MTF1, GLS, and CDKN2A were associated positively with 1 or more immune cell infiltrations in breast cancer. Patients with high levels of B cell, CD4+ T cell, CD8+ T cell, and dendritic cell infiltration had a higher survival rate at 10 years.

**Conclusion:**

This study comprehensively investigated relationships between cuproptosis and breast cancer by bioinformatic analyses. We found that cuproptosis-related genes were generally lowly expressed in breast carcinoma tissue. As the critical gene of cuproptosis, high expression of FDX1 was related to favorable prognoses in breast cancer patients; thus, it might be a potential prognostic marker. Moreover, genes associated with cuproptosis were linked to immune infiltration in breast cancer and this relationship affected the prognosis of breast cancer.

## 1. Introduction

With 2.3 million new cases estimated in 2022, breast cancer is the second most common cancer worldwide only after lung cancer.^[1]^ Moreover, its prevalence increases and gets younger every year, which brings a heavy burden to the world healthcare system.^[2]^ Recently, the effects of some special cell death forms, like ferroptosis, pyroptosis, necroptosis, and autophagy, in breast cancer have been investigated with meaningful results.^[3–6]^ As a newly discovered cell death form, cuproptosis was defined by Peter Tsvetkov et al in 2022.^[7]^ There is now a gap in cuproptosis and breast cancer research.

Cuproptosis is mediated by the copper ionophore, which is dependent on the accumulation of intracellular copper.^[7]^ When copper combines with the tricarboxylic acid (TCA) cycle proteins lipoylated, this leads to the clustering of lipoylated proteins and the loss of iron-sulfur cluster proteins, ultimately causing proteotoxic stress and cell death.^[7]^ This process is closely related to mitochondrial respiration, and the critical gene FDX1 can upregulate protein lipoylation and mitochondrial respiration to enhance cuproptosis.^[7]^ Valentina Oliveri proposed that some copper ionophores can act selectively on tumor cells, thus preferentially leading to cuproptosis of tumor cells.^[8]^ Serum copper levels of tumor patients, including breast cancer, differ markedly from those of normal subjects, and such variations may increase cancer risk.^[9–14]^ However, as a novel cell death, the effect of cuproptosis on breast cancer is unclear. Studies of cuproptosis-related genes may help reveal novel biological mechanisms in breast cancer carcinogenesis and progress and define new diagnostic, prognostic markers, and therapeutic targets. Studies in this area are imperative with the global burden of breast cancer.

Ten cuproptosis-related genes are known, including 7 up-regulated (FDX1, LIAS, LIPT1, DLD, DLAT, PDHA1, and PDHB) and 3 down-regulated genes (MTF1, GLS, and CDKN2A).^[7]^ This study probed the effects of genes associated with cuproptosis in breast invasive carcinoma (BRCA) patients comprehensively. We primarily analyzed cuproptosis-related gene expression levels, prognostic value, mutations, drug sensitivity, and immunocyte infiltration in BRCA. We performed multifaceted, multidimensional assessments for cuproptosis-related genes in breast cancer to identify their diagnosis, treatment, and prognosis value.

## 2. Methods

### 2.1. Data availability statement

The data analyzed in this study are open-source data. Readers can access them from the database links mentioned in the text and supplementary material.

### 2.2. Expression analysis

TIMER (https://cistrome.shinyapps.io/timer/), as a powerful and comprehensive tool, can perform the multifaceted evaluation of multiple tumors by TCGA (https://cancergenome.nih.gov/) data.^[15]^ Its “DiffExp module” is particularly prominent, being applied to evaluate relevant genes differentially expressed. In the present study, its “DiffExp module” was taken to analyze the cuproptosis-related gene expression in different tumors compared to normal tissues.

UALCAN (http://ualcan.path.uab.edu/index.html) is a functional internet instrument designed to analyze cancer OMICS data.^[16]^ We utilized it to identify whether cuproptosis-related genes are aberrantly expressed in BRCA relative to normal breast tissue. UALCAN is linked to TCGA, and the data analyzed above include 1097 BRCA tissues and 114 paraneoplastic tissues.

### 2.3. Survival prognosis analysis

Kaplan–Meier plotter (http://kmplot.com/analysis/) is a robust web-based tool focused on evaluating tumor prognosis.^[17]^ It mainly analyzes sequencing and gene microarray data of gene expression omnibus (GEO), EGA, and TCGA databases. We used it mainly for the analyses of the correlation of cuprotium-related genes with prognoses in BRCA patients; notably, it can analyze mRNA sequencing and microarray data. The sequencing data is mainly obtained from TCGA and GEO, and the sample size is larger than the TCGA sequencing data alone, so the results are more reliable. Analysis of cuproptosis-related gene expression and breast cancer survival were performed according to risk ratio (HR) and log-rank *P*-value. We also analyzed the chip data in the Kaplan–Meier plotter, and the probe set was selected as the “only JetSet best probe set.”

### 2.4. Gene expression analysis based on clinicopathological parameters

In addition, users can use clinical patient data to classify primary tumor samples and generate box plots for each gene expression level in each subgroup by UALCAN.^[16]^ Cuproptosis-related gene expression of BRCA patients with different individual cancer stages and molecular subtypes was analyzed by UALCAN.

### 2.5. Validation of gene expression

We used the datasets GSE20685, GSE42568, GSE29431, GSE45827, and GSE20711, including 703 BRCA tissues and 42 normal breast tissues, from GEO (https://www.ncbi.nlm.nih.gov/geo/) to validate the conclusions drawn from the TCGA data by comparing the cuproptosis-related gene expression of BRCA and normal breast. We downloaded the raw data as MINiML files and used the Wilcox test to assess differential gene expression. Box plots are drawn by “boxplot.”

The Human Protein Atlas (HPA) (https://www.proteinatlas.org) is a public database including immunohistochemistry-based expression data.^[18]^ All its data is open source. This paper used it to validate the cuproptosis-related molecule expression levels in breast cancer based on TCGA data.

### 2.6. Gene mutation analysis

The cBioPortal (www.cbioportal.org), as a feature-rich online database, contains large-scale cancer genomics datasets to analyze cancer genomics data in multiple dimensions.^[19]^ We used it for cuproptosis-related gene alteration analysis to obtain alteration frequencies and major alteration types. One thousand eighty-two breast cancer samples from TCGA were analyzed, including mutations, structural variant, putative copy-number alterations from GISTIC, and mRNA. Expression z-scores relative to diploid samples (RNASeq V2 RSEM) with a z-score threshold ± 1.8. Correlation of cuproptosis-related gene changes with overall survival (OS), progression-free survival (PFS), disease-specific survival (DSS), and disease-free survival in BRCA patients using Kaplan–Meier plots. The R package “pheatmap” presented the multigene correlation map.^[20]^

### 2.7. Interaction networks and enrichment analysis

STRING (https://string-db.org/), as a databank, contains massive amounts of data on protein–protein interactions and allows the construction of visual networks.^[21]^ We built an interaction network based on cuproptosis-related molecules using STRING; active interaction sources included text mining, experiments, databases, co‑expression, neighborhood, gene fusion, and co‑occurrence. A moderate confident level (0.400) of lowest interaction rating required for PPI networks was set.

To complement the PPI network mapped by STRING, we also employed the GeneMANIA Database (http://www.genemania.org), a genetic network integration instrument, generates and predicts gene interactions and visualizes interaction networks.^[22]^ Researchers utilized it for visualizations of the gene–gene interaction network regarding cuproptosis-related genes in prediction, physical interaction, pathways, co-expression, genetic interaction, and co-location.

DAVID (https://david.ncifcrf.gov/) serves as a functional commentary tool that enables users to make sense of the biological meaning behind their submitted gene lists.^[23]^ We used it for the gene ontology (GO) and Kyoto encyclopedia of genes and genomes (KEGG) enrichment analysis of cuproptosis-related genes. Visualization was accomplished through Sangerbox, an online free tool (http://www.sangerbox.com/tool).

### 2.8. Prediction of chemosensitivity

According to Genomics of Drug Sensitivity in Cancer (https://www.cancerrxgene.org/), the most prominent public pharmacogenomics database,^[24]^ we predicted the reaction to chemotherapy per TCGA sample. The above predictions were performed with the R package “pRRophetic.”^[25,26]^ The samples’ half-maximal inhibitory concentration (IC50) was assessed by ridge regression. We set all parameters to default values and used the batch effect of combat and tissue type of all tissues; the repeated gene expression was summarized as the mean value. The correlation of IC50 of chemotherapeutic agents with the cuproptosis-related gene expression levels was investigated using Spearman correlation analysis. The “ggstatsplot” package mapped the results.^[27]^

### 2.9. Association of genes expression with immune infiltration and immune checkpoints

TIMER is not only able to perform gene expression analysis, but its immune cell infiltration analysis is even better.^[28]^ Its “Gene Module” was employed to analyze and visualize the correlation between cuproptosis-related gene expression levels and infiltration levels of 6 immune cell types (B cell, CD4+ T cell, CD8+ T cell, neutrophil, macrophage, and dendritic cell) in BRCA. We also utilized the “Survival Module” to assess the impact on the prognosis in BRCA patients by immune infiltration.

The correlation of common immune checkpoint genes with genes associated with cuproptosis was also analyzed.^[29–31]^ The visualization was achieved with “pheatmap.”^[32]^ For predicting the antitumor effect of immunosuppressive agents, we further analyzed the correlation of expression levels of cuproptosis-related genes with tumor mutation burden (TMB) and microsatellite instability (MSI) using the “ggstatsplot” package of R software.^[27]^

### 2.10. Statistical analysis

We used online tools (TIMER, UALCAN, Kaplan–Meier plotter, HPA, cBioPortal, STRING, GeneMANIA, and DAVID) and R program v4.0.3 for statistical analyses and visualizations. The Wilcox test and the Student *t* test were adopted to assess gene expression levels in cancers and normal tissues. Log-rank test was performed to determine the correlation of gene expression with prognosis in BRCA patients. The correlation of cuproptosis-related gene expression levels with chemotherapy drugs’ IC50 and TMB/MSI was probed by spearman correlation analysis. As for spearman correlation analyses, correlation is higher the closer in absolute value the *R*_s_ approaches 1. The correlation can be graded according to the absolute value of *R*_s_ as follows: <0.10, 0.10–0.39, 0.40–0.69, 0.70–0.89, 0.90–1.00 are defined as negligible correlation, weak correlation, moderate correlation, strong correlation, and very strong correlation, respectively. For all analyses in this paper, *P* < .05 is statistically significant; *P < .05, **P < .01, ***P < .001, except as noted otherwise.

## 3. Results

### 3.1. Aberrant mRNA expression of genes associated with cuproptosis in tumors

**Figure 1.**
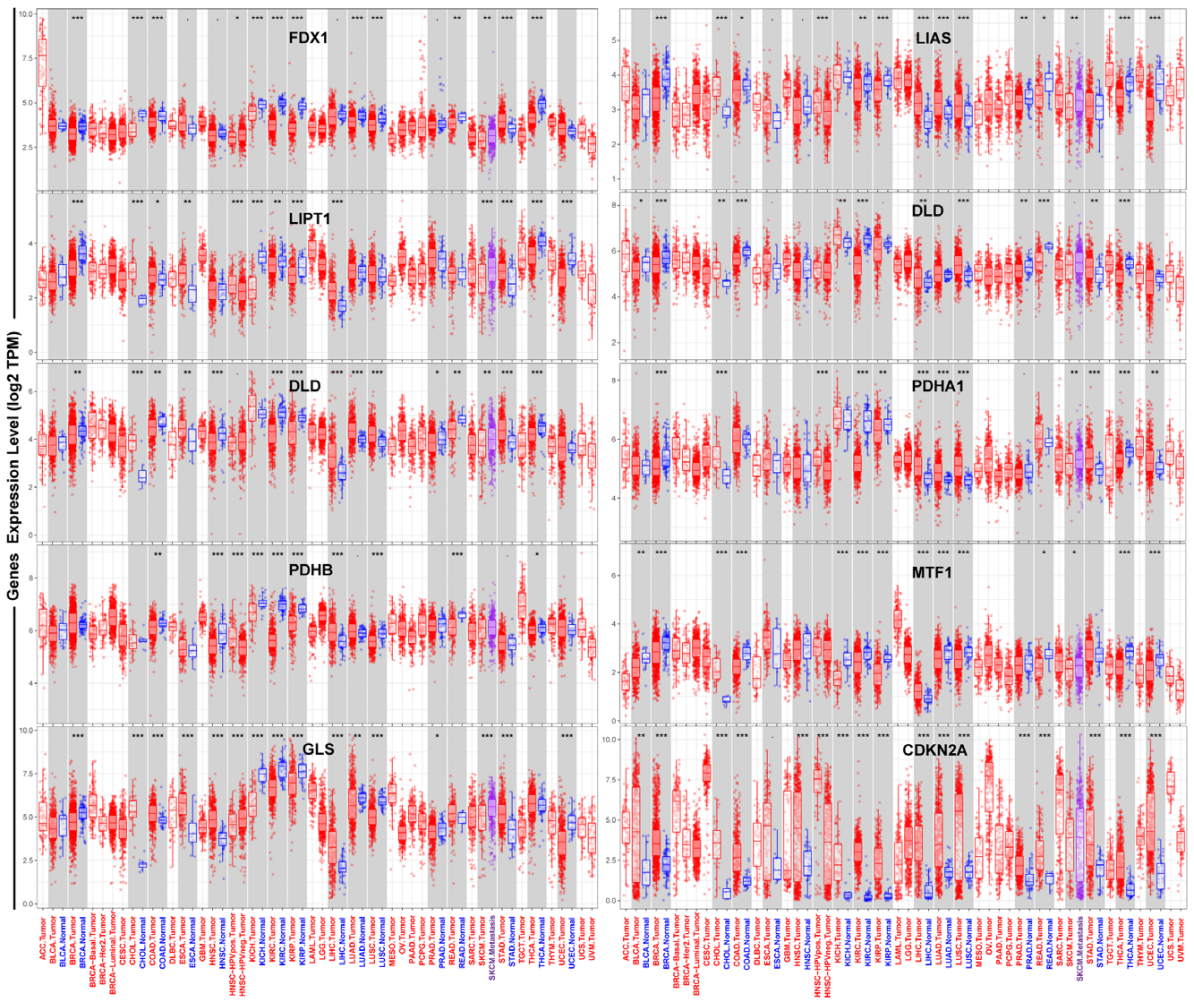
Cuproptosis-related gene expression in various human cancers and normal tissues (TIMER).

**Figure 2.**
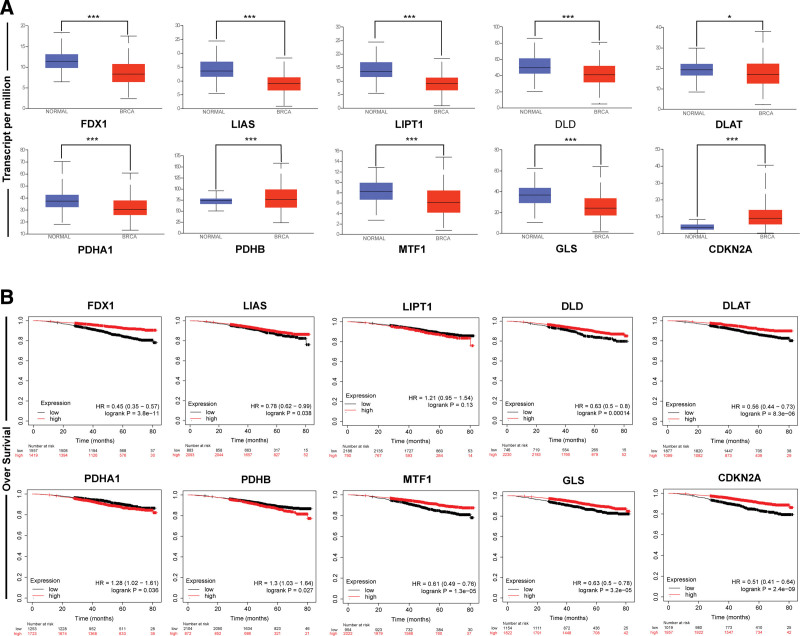
Cuproptosis-related genes expression breast cancer and its prognostic value. (A) FDX1, LIAS, LIPT1, DLD, DLAT, PDHA1, MTF1, and GLS were downregulated in breast cancer, while PDHB and CDKN2A were upregulated (UALCAN). (B) FDX1, LIAS, DLD, DLAT, MTF1, GLS, and CDKN2A were associated with longer OS, but the higher expression of PDHA1 and PDHB were associated with poorer OS (Kaplan–Meier plotter, mRNA sequencing data). OS = overall survival.

TIMER was utilized to identify the aberrant expression of cuproptosis-related genes at the transcriptional level in pan-cancerous tissues compared with respective normal samples. As shown in Figure 1, cuproptosis core gene FDX1 was down-regulated in BRCA, cholangiocarcinoma, colorectal adenocarcinoma, kidney chromophobe carcinoma, kidney renal clear cell carcinoma, kidney renal papillary cell carcinoma, lung adenocarcinoma, lung squamous cell carcinoma, rectal adenocarcinoma, thyroid carcinoma but up-regulated in stomach adenocarcinoma. The other genes were generally down-regulated in multiple tumors relative to corresponding normal subjects (Fig. 1). Table S1, Supplemental Digital Content, http://links.lww.com/MD/N747 provides *P* values of TIMER analysis. For BRCA, all cuproptosis-related genes except PDHB were aberrantly expressed. However, in the UALCAN cohort, expression of all cuproptosis-related genes was abnormal in BRCA. FDX1 (*P* < 1E‐12), LIAS (*P* = 2.22E‐16), LIPT1 (*P* < 1E‐12), DLD (*P* < 5.46E‐09), DLAT (*P* = 3.14E‐02), PDHA1 (*P* = 1.15E‐07), MTF1 (*P* = 1.07E‐09), and GLS (*P* = 2.48E‐05) were downregulated in breast cancer, while PDHB (*P* = 5.04E‐07) and CDKN2A (*P* = 1.62E‐12) were upregulated (Fig. 2A). Combining the TIMER and UALCAN results yielded FDX1, LIAS, LIPT1, DLD, DLAT, PDHA1, MTF1, and GLS were down-regulated in breast cancer tissues, but CDKN2A was up-regulated.

### 3.2. Correlation of cuproptosis-related genes with BRCA patients’ prognoses

The correlation of cuproptosis-related gene expression and OS in 2976 BRCA mRNA sequencing samples were displayed in Figure 2B. Only LIPT1 were not associated with OS. Moreover, higher transcript levels of FDX1, LIAS, DLD, DLAT, MTF1, GLS, and CDKN2A were associated with longer OS (FDX1: HR = 0.45, 95% CI = 0.35–0.57, *P* = 3.8E‐11; LIAS: HR = 0.78, 95% CI = 0.62–0.99, *P* = .038; DLD: HR = 0.63, 95% CI = 0.50–0.80, *P* = .00014; DLAT: HR = 0.56, 95% CI = 0.44–0.73, *P* = 8.3E‐06; MTF1: HR = 0.61, 95% CI = 0.49–0.76, *P* = 1.3E‐05; GLS: HR = 0.63, 95% CI = 0.50–0.78, *P* = 3.2E‐05; CDKN2A: HR = 0.51, 95% CI = 0.41–0.64, *P* = 2.4E‐09), while higher expression of PDHA1 and PDHB were related to shorter OS (PDHA1: HR = 1.28, 95% CI = 1.02–1.61, *P* = .036; PDHB: HR = 1.3, 95% CI = 1.03–1.64, *P* = .027).

**Figure 3.**
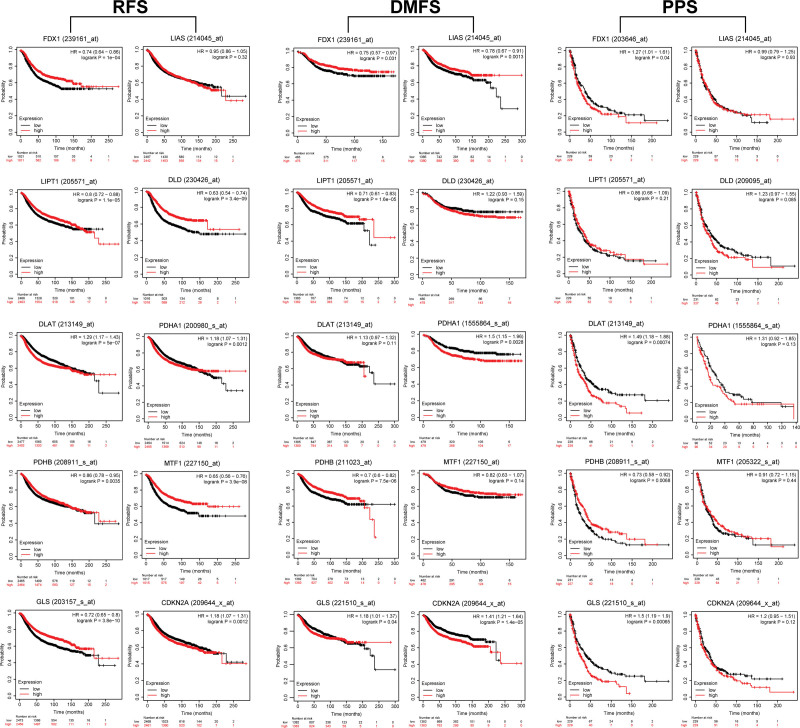
Correlation of cuproptosis-related genes expression levels with RFS, DMFS, and PPS in breast cancer patients (Kaplan–Meier plotter, mRNA microarray data). DMFS = distant metastasis-free survival, PPS = post-progressive survival, RFS = recurrence-free survival.

We further explored the effect of cuproptosis-related gene expression on recurrence-free survival (RFS), distant metastasis-free survival (DMFS), and post-progressive survival (PPS) in breast cancer by mRNA microarray data. The results were shown in Figure 3. As for RFS, all cuproptosis-related genes except LIAS was associated with RFS. High transcript levels of FDX1, LIPT1, DLD, PDHB, MTF1, and GLS were associated with favorable RFS (FDX1: HR = 0.74, 95% CI = 0.64–0.86, *P* = 1.0E‐04; LIPT1: HR = 0.80, 95% CI = 0.72–0.88, *P* = 1.1E‐05; DLD: HR = 0.63, 95% CI = 0.54–0.74, *P* = 3.4E‐09; PDHB: HR = 0.86, 95% CI = 0.78–0.95, *P* = .0035; MTF1: HR = 0.65, 95% CI = 0.56–0.76, *P* = 3.9E‐08; GLS: HR = 0.72, 95% CI = 0.65–0.80, *P* = 3.8E‐10), while analysis of DLAT, PDHA1, and CDKN2A yielded the opposite results (DLAT: HR = 1.29, 95% CI = 1.17–1.43, *P* = 5E‐07; PDHA1: HR = 1.18, 95% CI = 1.07–1.31, *P* = .0012; CDKN2A: HR = 1.18, 95% CI = 1.07–1.31, *P* = .0012). Patients with increased expression of FDX1, LIAS, LIPT1, and PDHB had longer DMFS (FDX1: HR = 0.75, 95% CI = 0.57–0.97, *P* = .031; LIAS: HR = 0.78, 95% CI = 0.67–0.91, *P* = .0013; LIPT1: HR = 0.71, 95% CI = 0.61–0.83, *P* = 1.6E‐05; PDHB: HR = 0.70, 95% CI = 0.60–0.82, *P* = 7.5E‐06), while the results of PDHA1, GLS, and CDKN2A were opposite. (PDHA1: HR = 1.5, 95% CI = 1.15–1.96, *P* = .0028; GLS: HR = 1.18, 95% CI = 1.01–1.37, *P* = .04; CDKN2A: HR = 1.41, 95% CI = 1.21–1.64, *P* = 1.4E‐05). As for PPS, higher expression of FDX1, DLAT, and GLS were related to poorer PPS (FDX1: HR = 1.27, 95% CI = 1.01–1.61, *P* = .04; DLAT: HR = 1.49, 95% CI = 1.18–1.88, *P* = .00074; GLS: HR = 1.50, 95% CI = 1.19–1.90, *P* = .00065), while the increased PDHB expression level related to longer PPS (PDHB: HR = 0.73, 95% CI = 0.58–0.92, *P* = .0068).

### 3.3. Differential expression of cuproptosis-related genes based on different tumor stages and subtypes in BRCA patients

**Figure 4.**
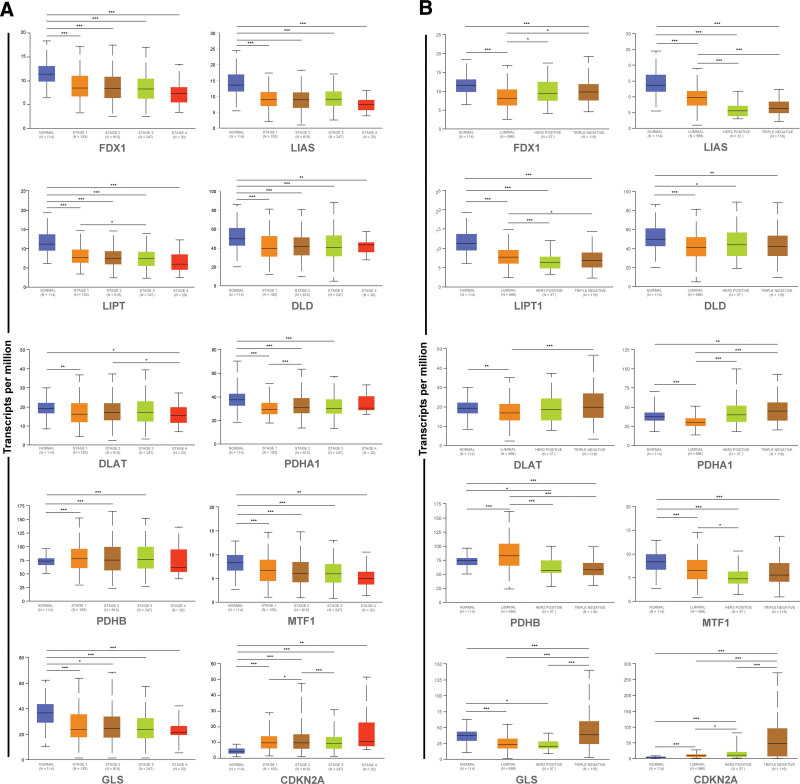
Differential expression of genes associated with cuproptosis among breast cancer patients with different individual cancer stages and molecular subtypes. (A) Individual cancer stage. (B) Molecular subtype.

Cuproptosis-related gene expression levels among BRCA patients with different individual cancer stages and molecular subtypes were investigated using UALCAN, and the results were shown in Figure 4. Differential expression between normal tissues and BRCA tissues at each tumor stage was found in almost all cuproptosis-associated genes; however, only LIPT1, DLAT, PDHA1, and CDKN2A were differentially expressed among tumor stages (Fig. 4A). PDHA1 and CDKN2A were differentially expressed in stage I and stage II (PDHA1: *P* = 2.248E‐04; CDKN2A: *P* = .035). Differential expression of LIPT1, CDKN2A, and DLAT was present in stage I versus stage III, stage II versus stage III, and stage II versus stage IV, respectively (LIPT1: *P* = .011; CDKN2A: *P* = 9.559E‐04; DLAT: *P* = 4.998E‐02). As for the molecular subtype, differential expressions of cuproptosis-related genes were found between normal tissues and different subtypes of BRCA, what is more, among the various subtypes (Fig. 4B). FDX1, LIAS, LIPT1, PDHA1, PDHB, MTF1, and CDKN2A were differentially expressed in Luminal and Her-2 positive (FDX1: *P* = .021; LIAS: *P* = 3.195E‐09; LIPT1: *P* = 1.213E‐04; PDHA1: *P* = 5.954E‐04; PDHB: *P* = 2.150E‐05; MTF1: *P* = .024; CDKN2A: *P* = .038). The genes differentially expressed in Luminal and Triple-negative were FDX1, LIAS, LIPT1, DLAT, PDHA1, PDHB, GLS, and CDKN2A (FDX1: *P* = 1.959E‐06; LIAS: *P* = 3.195E‐09; LIPT1: *P* = .023; DLAT: *P* = 2.415E‐04; PDHA1: *P* = 8.549E‐15; PDHB: *P* < 1E‐12; GLS: *P* = 3.232E‐09; CDKN2A: *P* = 4.701E‐12). Only GLS and CDKN2A were differentially expressed in Her-2 positive and Triple-negative (GLS: *P* = 9.615E‐07; CDKN2A: *P* = 2.266E‐06).

### 3.4. Validation of the expression levels of cuproptosis-related genes in BRCA patients

**Figure 5.**
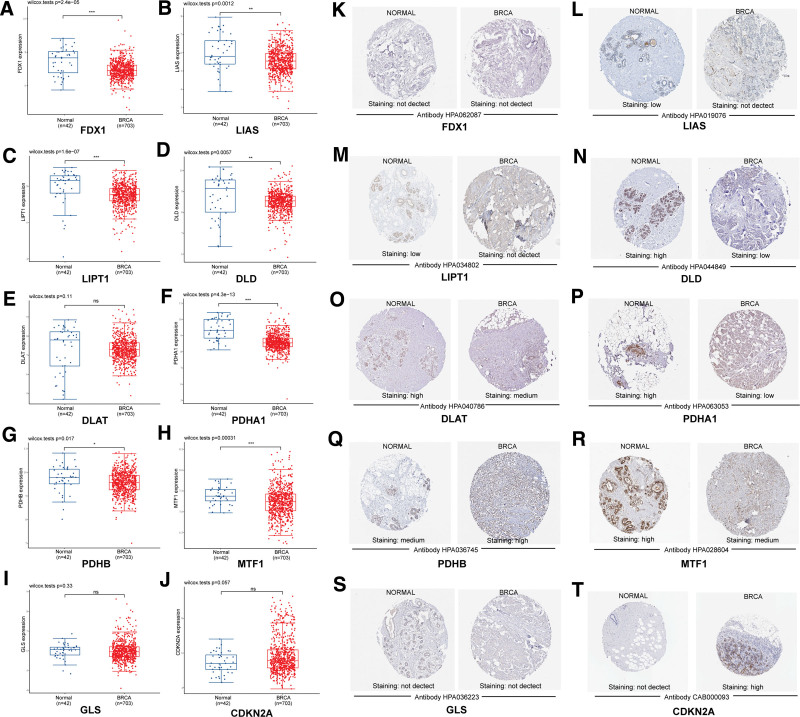
Validation of cuproptosis-related gene expression in breast cancer compared with normal tissues by GEO data and immunohistochemistry. (A–J) FDX1, LIAS, LIPT1, DLD, PDHA1, MTF1 and PDHB were down-regulated in breast cancer, while DLAT, GLS, and CDKN2A were not differentially expressed in breast cancer and normal tissues (GEO data). (K–T) LIAS, LIPT, DLD, DLAT, PDHA1, and MTF1 proteins were expressed down-regulated in breast cancer, while PDHB and CDKN2A were expressed up-regulated in BRCA, and the expression of FDX1 and GLS molecules was not different in breast cancer compare with normal tissues (HPA). BRCA = breast invasive carcinoma, GEO = gene expression omnibus, HPA = Human Protein Atlas.

For the validation of the gene expression results derived from TCGA data, we downloaded and analyzed the GEO data (GSE20685, GSE42568, GSE29431, GSE45827, and GSE20711) containing 703 breast cancer tissues and 42 normal breast tissues. Table 1 provides clinicopathological information for TCGA and GEO data. After analyzing the GEO data, We found that FDX1, LIAS, LIPT1, DLD, PDHA1, PDHB, and MTF1 were expressed lower in BRCA than in normal breast tissues (FDX1: *P* = 2.4E‐05; LIAS: *P* = .0012; LIPT1: *P* = 1.6E‐07; DLD: *P* = .0057; PDHA1: *P* = 4.3E‐13; PDHB: *P* = .017; MTF1: *P* = .00031), but DLAT, GLS, and CDKN2A were not differentially expressed between the 2 groups (DLAT: *P* = .11, GLS: *P* = .33, CDKN2A: *P* = .057) (Fig. 5A–J).

**Table 1:** Clinicopathological information of TCGA data and GEO data.

		TCGA	GSE20685	GSE42568	GSE29431	GSE45827	GSE20711
*No. of patients*		1097	327	104	54	130	88
*Status*	Alive	943	244	69	NA	NA	NA
	Dead	154	83	35	NA	NA	NA
*Age*	Median [Min, Max]	58 [26, 90]	46 [24,84]	30 [31, 89]	51.5 [36, 82]	NA	NA
*Gender*	Female	1085	327	104	54	130	88
	Male	12	0	0	0	0	0
*Race*	American Indian	1	NA	NA	NA	NA	NA
	Asian	61	NA	NA	NA	NA	NA
	Black	182	NA	NA	NA	NA	NA
	White	759	NA	NA	NA	NA	NA
*Major subclasses*	Luminal	566	NA	67	NA	59	35
	HER-2 positive	37	NA	NA	28	30	26
	Triple-negative	166	NA	NA	NA	41	27
*pTNM-stage*	I	89	NA	NA	NA	NA	NA
	IA	86	NA	NA	NA	NA	NA
	IB	6	NA	NA	NA	NA	NA
	II	6	NA	NA	NA	NA	NA
	IIA	359	NA	NA	NA	NA	NA
	IIB	259	NA	NA	NA	NA	NA
	III	2	NA	NA	NA	NA	NA
	IIIA	154	NA	NA	NA	NA	NA
	IIIB	27	NA	NA	NA	NA	NA
	IIIC	66	NA	NA	NA	NA	NA
	IV	20	NA	NA	NA	NA	NA
*Individual cancer stages*	Stage1	183	137	NA	3	NA	13
	Stage2	615	87	NA	11	NA	5
	Stage3	247	63	NA	25	NA	70
	Stage4	20	40	NA	0	NA	0

GEO = gene expression omnibus.

The immunohistochemistry results of cuproptosis-related genes in breast cancer were shown in Figure 5K–T. LIAS, LIPT, DLD, DLAT, PDHA1, and MTF1 were expressed higher than BRCA in normal tissues, while the opposite was true for PDHB and CDKN2A, and the expression of FDX1 and GLS molecules was not different in BRCA compare with normal tissues.

### 3.5. Genetic alteration in cuproptosis-related genes and prognostic value in BRCA patients

**Figure 6.**
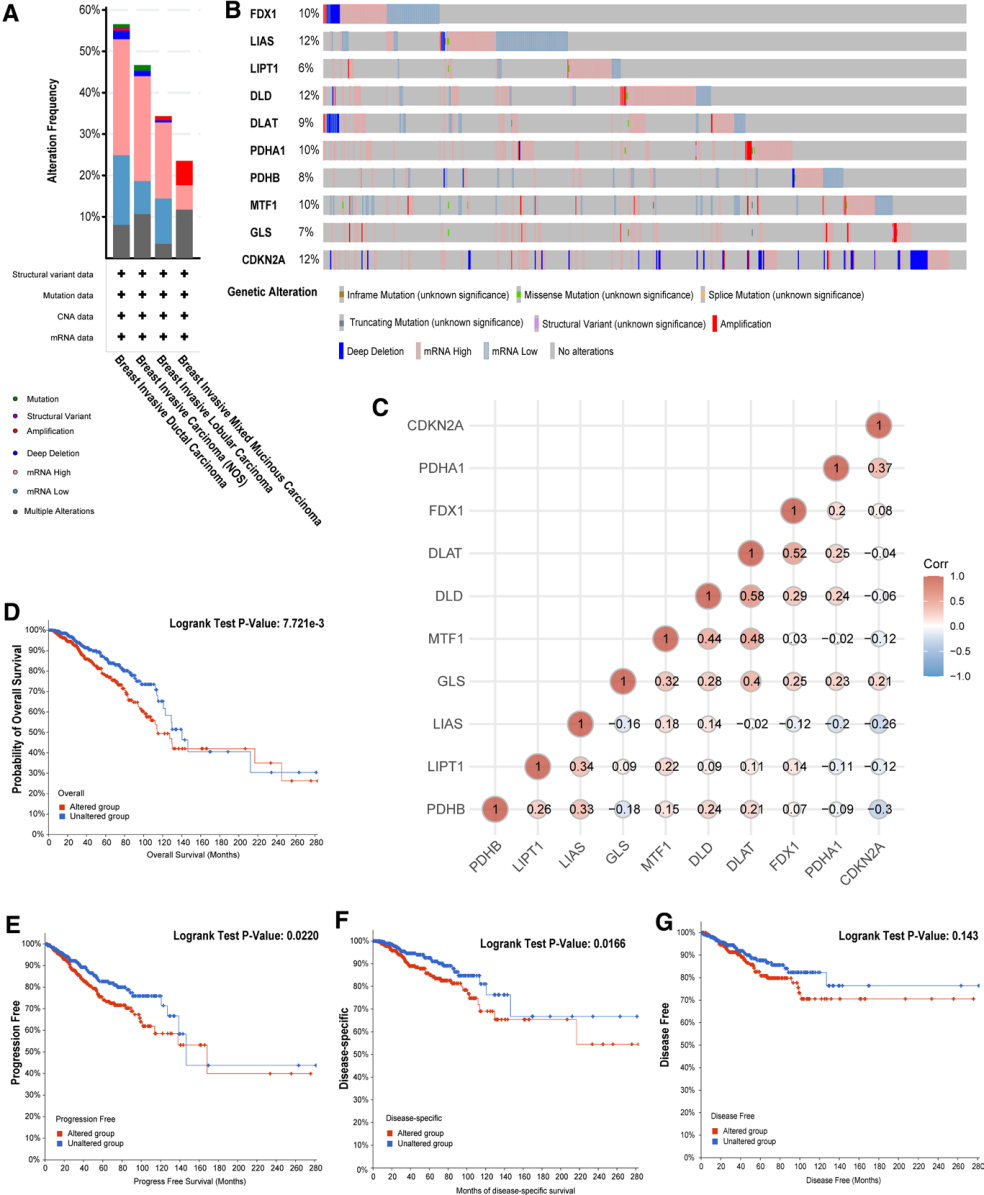
Genetic alterations in cuproptosis-related genes in BRCA patients and their prognostic values (cBioPortal). (A) Summary of cuproptosis-related gene mutations in breast cancer. (B) Cuproptosis-related genes have a high mutation rate (51.3%) in breast cancer, with LIAS, DLD, and CDKN2A having the highest mutation frequency, all at 12%, while LIPT1 had the lowest mutation rate at 6%. (C) Relationship between different cuproptosis-related genes. (D–G) Mutant cuproptosis-related genes were related to worse OS, PFS, and DSS but not to DFS. DFS = disease-free survival, DSS = disease-specific survival, OS = overall survival, PFS = progression-free survival.

We used cBioPortal to explore the status of mutations in cuproptosis-associated genes based on 1082 breast cancer samples and conducted prognostic analysis. The mutations were mainly concentrated in breast invasive ductal carcinoma, while the alteration frequency was the lowest in breast invasive mixed mucinous carcinoma; enhanced mRNA expression was the most frequent mutation (Fig. 6A). The mutation frequency of cuproptosis-related genes in BRCA was 51.3% (Fig. 6B). LIAS, DLD, and CDKN2A had the highest variant frequencies in all cuproptosis-related genes, all at 12%, while LIPT1 was 6%, the lowest mutation rate. The correlation between genes associated with cuproptosis were provided in Figure 6C, DLAT was highly positively related to FDX1 (*P* < .05, Cor = 0.516) and DLD (*P* < .05, Cor = 0.584). Further, the Kaplan–Meier plots showed cuproptosis-related gene mutations related to poorer OS, PFS, and DSS (OS: *P* = 7.721E‐3; PFS: *P* = .0220; DSS: *P* = .0166) (Fig. 6D–G).

### 3.6. Interaction networks and enrichment analyses

**Figure 7.**
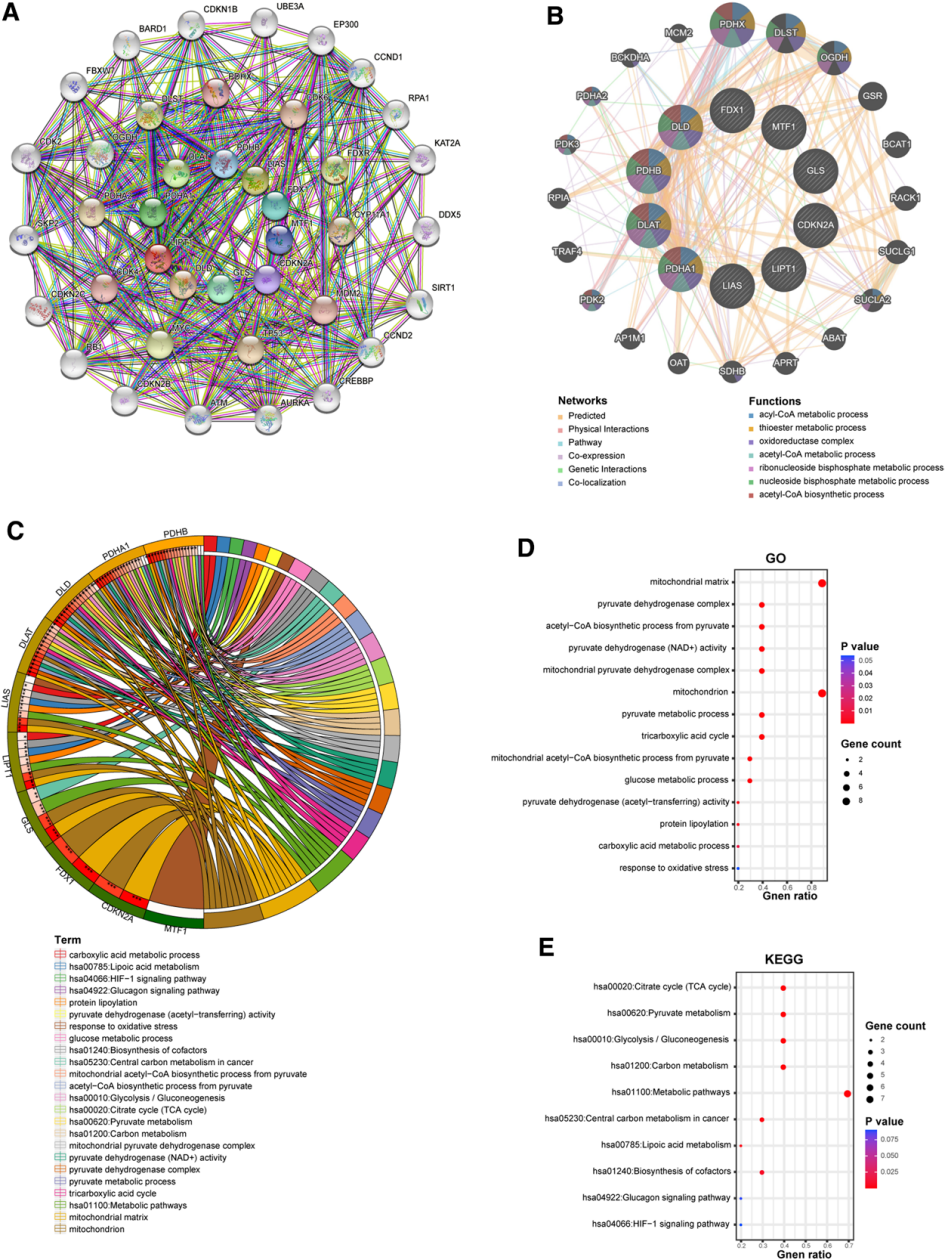
Interaction networks and GO, KEGG enrichment analyses. (A) PPI network of cuproptosis-related molecules. (B) Gene–gene interaction network of cuproptosis-related genes. (C–E) Results of the enrichment analysis of cuproptosis-related genes.

The PPI network of 10 genes regarding cuproptosis was mapped by STRING and obtained 40 nodes with 259 edges (Fig. 7A), of which there were 99 expected edges. We also obtained 10 genes (PDHX, CDK4, MDM2, CDK6, CYP11A1, TP53, PDHA2, DLST, FDXR, and MYC) that were highly functionally related to cuproptosis-related genes. Figure 7B showed the gene–gene interaction network of 10 cuproptosis-related genes produced by GeneMANIA, including the 10 cuproptosis-related genes and the 20 genes that interacted most significantly. The network includes prediction, physical interaction, pathways, co-expression, genetic interaction, and co-location. DLD, PDHB, DLAT, and PDHA1 all correlated with acyl-CoA metabolic process, thioester metabolic process, oxidoreductase complex, acetyl-CoA metabolic process, ribonucleoside bisphosphate metabolic process, nucleoside bisphosphate metabolic process, acetyl-CoA biosynthetic process.

We completed GO and KEGG analysis for 10 genes regarding cuproptosis by DAVID. The results are provided in Figure 7C–E, and Table 2. Thirteen GO terms were associated with cuproptosis-related genes, which could be divided into 3 functional groups: molecular function, biological process, and cellular component. Eight KEGG pathways were correlated with cuproptosis-related genes.

**Table 2:** Results of GO and KEGG enrichment analysis of cuproptosis-related genes.

Term	Description	Gene count	*P*-value	FDR
*MF*				
GO:0034604	Pyruvate dehydrogenase (NAD+) activity	4	1.52E‐09	6.09E‐08
GO:0004739	Pyruvate dehydrogenase (acetyl-transferring) activity	2	.001438	0.028757
*BP*				
GO:0006086	Acetyl-CoA biosynthetic process from pyruvate	4	1.39E‐09	1.54E‐07
GO:0006090	Pyruvate metabolic process	4	1.07E‐07	5.92E‐06
GO:0006099	Tricarboxylic acid cycle	4	5.36E‐07	1.98E‐05
GO:0061732	Mitochondrial acetyl-CoA biosynthetic process from pyruvate	3	1.92E‐06	5.33E‐05
GO:0006006	Glucose metabolic process	3	3.70E‐04	0.008217
GO:0009249	Protein lipoylation	2	.002787958	0.051577231
GO:0019752	Carboxylic acid metabolic process	2	.010649692	0.168873686
GO:0006979	Response to oxidative stress	2	.054020261	0.74953112
*CC*				
GO:0005759	Mitochondrial matrix	9	1.52E‐13	2.73E‐12
GO:0045254	Pyruvate dehydrogenase complex	4	6.15E‐10	5.53E‐09
GO:0005967	Mitochondrial pyruvate dehydrogenase complex	4	3.44E‐09	2.06E‐08
GO:0005739	Mitochondrion	9	4.90E‐09	2.21E‐08
*KEGG*				
hsa00020	Citrate cycle (TCA cycle)	4	1.58E‐06	5.85E‐05
hsa00620	Pyruvate metabolism	4	6.28E‐06	1.16E‐04
hsa00010	Glycolysis/gluconeogenesis	4	1.84E‐05	2.27E‐04
hsa01200	Carbon metabolism	4	9.32E‐05	8.62E‐04
hsa01100	Metabolic pathways	7	2.65E‐04	0.001962834
hsa05230	Central carbon metabolism in cancer	3	.001499018	0.009243944
hsa00785	Lipoic acid metabolism	2	.003447848	0.01822434
hsa01240	Biosynthesis of cofactors	3	.006974459	0.032256873
hsa04922	Glucagon signaling pathway	2	.088789482	0.334415278
hsa04066	HIF-1 signaling pathway	2	.090382508	0.334415278

BP = biological process, CC = cellular component, MF = molecular function.

### 3.7. Relationship between chemosensitivity and genes associated with cuproptosis in BRCA

**Figure 8.**
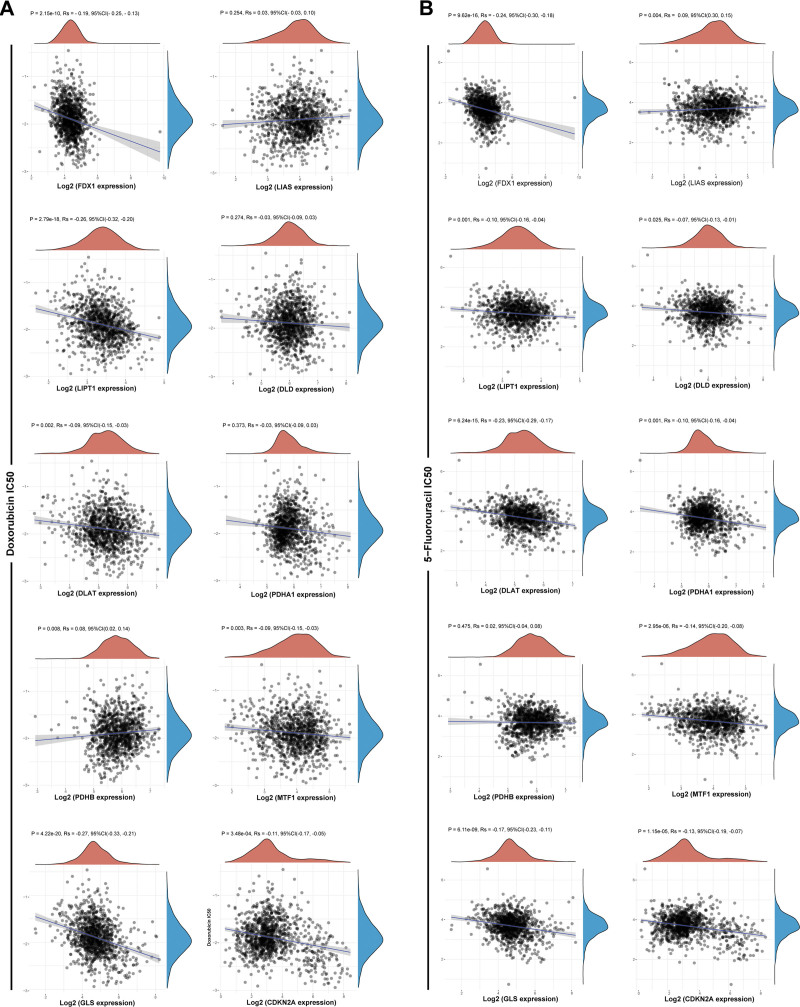
Correlation of cuproptosis-related genes. Expression values with IC50 of doxorubicin and 5-fluorouracil. (A) The expression of FDX1, LIPT1, GLS, and CDKN2A were negatively correlated with IC50 of doxorubicin. (B) FDX1, LIPT1, DLAT, PDHA1, MTF1, GLS, and CDKN2A expression was negatively correlated with IC50 of 5-fluorouracil. IC50 = disease-free survival.

The relationship between the IC50 of conventional chemotherapeutic agents (doxorubicin, 5-fluorouracil, docetaxel, and cisplatin) and cuproptosis-related gene expression in BRCA patients was assessed to predict chemosensitivity. The lower the IC50 of a drug indicates that the drug is more responsive to the sample and the better the treatment effect. For doxorubicin, the expression of FDX1, LIPT1, GLS, and CDKN2A were all negatively correlated with IC50 (FDX1: *P* = 2.15E‐10, *R*_s_ = ‐0.19; LIPT1: *P* = 2.79E‐18, *R*_s_ = ‐0.26; GLS: *P* = 4.22E‐20, *R*_s_ = ‐0.27; CDKN2A: *P* = 3.48E‐04, *R*_s_ = ‐0.11) (Fig. 8A). For 5-fluorouracil, the expression of FDX1, LIPT1, DLAT, PDHA1, MTF1, GLS, and CDKN2A was negatively correlated with IC50 (FDX1: *P* = 9.62E‐16, R_s_ = ‐0.24; LIPT1: *P* = .001, R_s_ = ‐0.10; DLAT: *P* = 6.24E‐15, R_s_ = ‐0.23; PDHA1: *P* = .001, *R*_s_ = ‐0.10; MTF1: *P* = 2.95E‐06, *R*_s_ = ‐0.14; GLS: *P* = 6.11E‐09, *R*_s_ = ‐0.17; CDKN2A: *P* = 1.15E‐05, *R*_s_ = ‐0.13) (Fig. 8B). For docetaxel, the expression levels of all 10 cuproptosis-related genes correlated with IC50. FDX1, DLD, DLAT, PDHA1, MTF1, GLS, and CDKN2A were negatively related to IC50 (FDX1: *P* = .001, *R*_s_ = ‐0.10; DLD: *P* = 1.4E‐07, *R*_s_ = ‐0.16; DLAT: *P* = 3.67E‐08, *R*_s_ = ‐0.17; PDHA1: *P* = 5.71E‐09, *R*_s_ = ‐0.17; MTF1: *P* = 4.71E‐08, *R*_s_ = ‐0.16; GLS: *P* = 8.46E‐18, *R*_s_ = ‐0.26; CDKN2A: *P* = 2.7E‐08, *R*_s_ = ‐0.17), while LIAS, LIPT1, and PDHB were the opposite (LIAS: *P* = 2.34E‐10, *R*_s_ = 0.19; LIPT1: *P* = 8.91E‐05, *R*_s_ = 0.12; PDHB: *P* = 2.54E‐06, *R*_s_ = 0.14) (Fig. S1A, Supplemental Digital Content, http://links.lww.com/MD/N747). For cisplatin, the expression values of FDX1, PDHA1, GLS, and CDKN2A were negatively related to IC50 (FDX1: *P* = 1.77E‐13, *R*_s_ = ‐0.22; PDHA1: *P* = 4.17E‐09, *R*_s_ = ‐0.18; GLS: *P* = 1.71E‐31, *R*_s_ = ‐0.34; CDKN2A: *P* = 2.27E‐26, *R*_s_ = ‐0.31), but the opposite outcomes were found in LIAS, LIPT1, PDHB, and MTF1 (LIAS: *P* = 4.75E‐43, *R*_s_ = 0.40; LIPT1: *P* = 2.43E‐08, *R*_s_ = 0.17; PDHB: *P* = 2.69E‐60, *R*_s_ = 0.47; MTF1: *P* = 9.95E‐05, *R*_s_ = 0.12) (Fig. S1B, Supplemental Digital Content, http://links.lww.com/MD/N747).

### 3.8. Correlation of cuproptosis-related genes with immunocyte infiltration in BRCA patients

**Figure 9.**
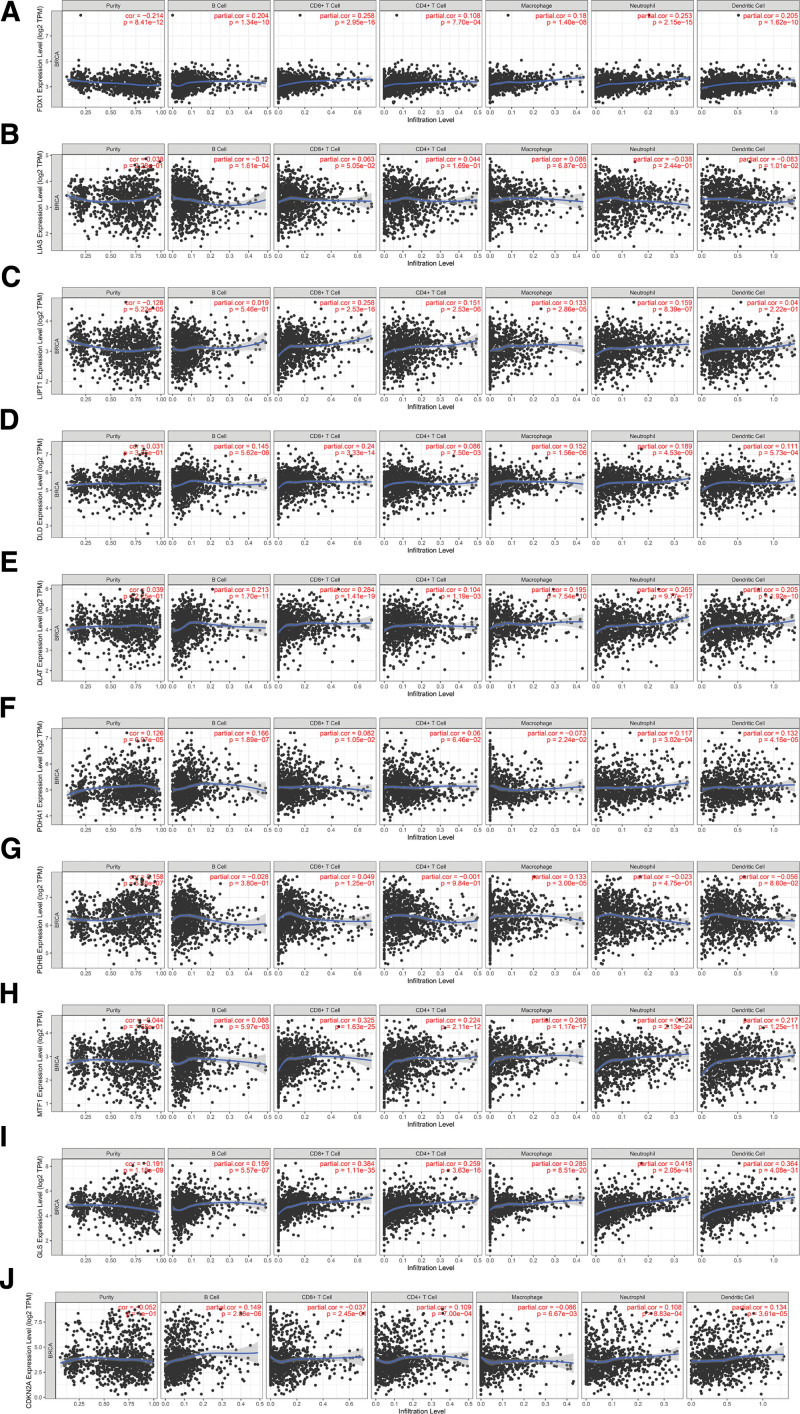
Relationship between cuproptosis-related genes and immunocyte infiltration in breast cancer patients (TIMER). (A) FDX1, (B) LIAS, (C) LIPT1, (D) DLD, (E) DLAT, (F) PDHA1, (G) PDHB, (H) MTF1, (I) GLS, (J) CDKN2A.

The association between cuproptosis-related gene expression values and immunocyte abundance in BRCA was shown in Figure 9. The FDX1, DLAT, and GLS expressions were positively associated with the abundance of all 6 immunocytes (Fig. 9A, E, and I). The expression value of LIAS was negatively associated with B cell infiltration (Fig. 9B). LIPT1 expression positively related to CD4+ T cells, CD8+ T cells, Neutrophils, and macrophages infiltration (Fig. 9C). DLD expression was positively associated with the infiltration of B cells, CD8+ T cells, neutrophils, macrophages, and dendritic cells (Fig. 9D). PDHA1 expression was actively associated with infiltration of B cells, neutrophils, and dendritic cells (Fig. 9F). The expression of PDHB was a positive correlation with macrophages infiltration (Fig. 9G). The expression value of MTF1 was positively related to the infiltration of CD4+ T cells, CD8+ T cells, neutrophils, macrophages, and dendritic cells (Fig. 9H). CDKN2A expression was positively related to the infiltration of B cells, CD4+ T cells, neutrophils, and dendritic cells (Fig. 9J). Table S2, Supplemental Digital Content, http://links.lww.com/MD/N747 provides the results.

The prognostic value of immune cell infiltration in BRCA was also analyzed. The immunocyte abundance was not related to cumulative survival at 5 years in BRCA patients (Fig. S2A, Supplemental Digital Content, http://links.lww.com/MD/N747). However, after investigating 10 years of data, we found that higher expression of B cells (*P* = .017), CD4+ T cells (*P* = .006), CD8+ T cells (*P* = .034), and dendritic cells (*P* = .001) related to longer cumulative survival (Fig. S2B, Supplemental Digital Content, http://links.lww.com/MD/N747).

### 3.9. Association between cuproptosis-related genes and immune checkpoints

**Figure 10.**
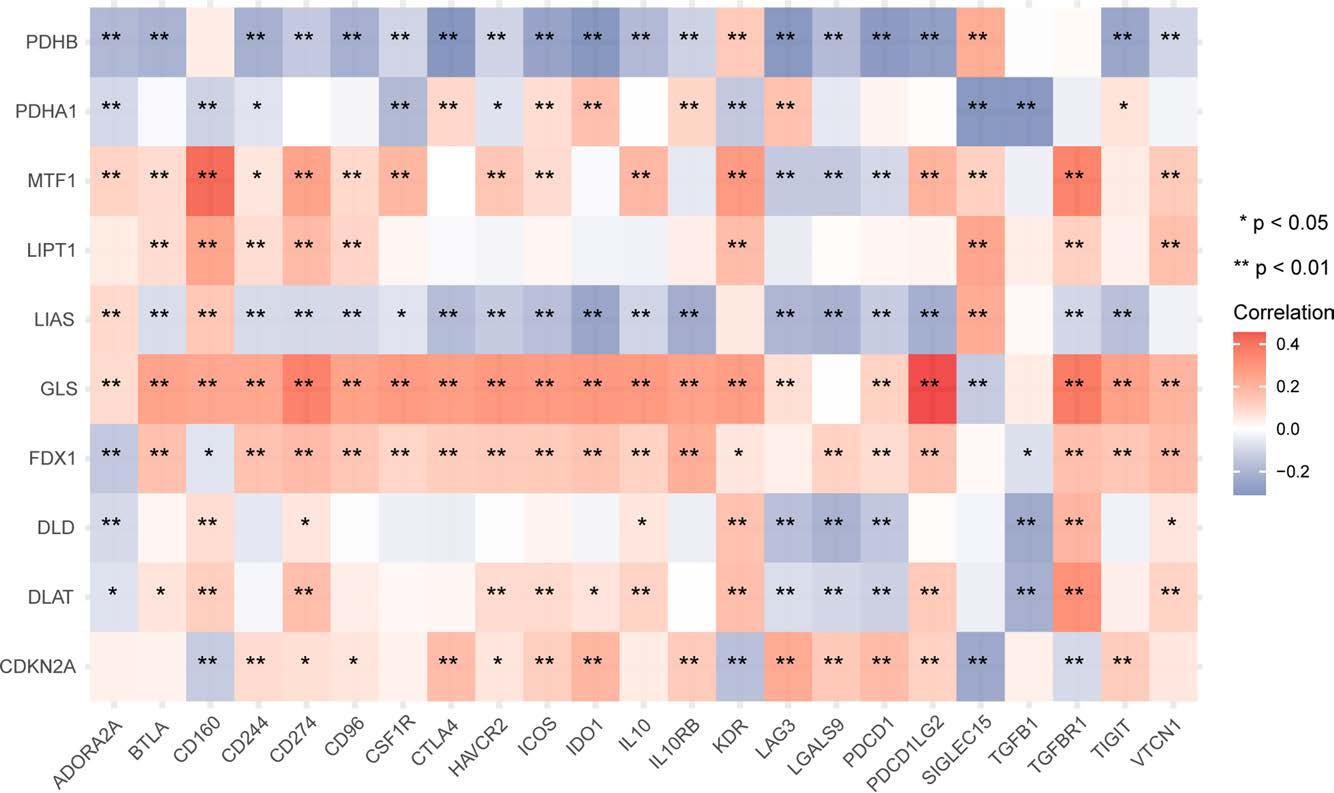
Correlation of cuproptosis-related genes with common immune checkpoint genes.

We also investigated their correlation with common immune checkpoints. The results are shown in Figure 10, the strongest correlation was found between GLS and the immune checkpoint gene PDCDILG2 (*P* < .05, Cor = 0.456). As for TMB/MSI, we showed that the expression of DLAT, PDHA1, GLS, and CDKN2A was positively related to TMB (DLAT: *P* = 2.03E‐04, *R*_s_ = 0.12; PDHA1: *P* = 2.12E‐26, *R*_s_ = 0.33; GLS: *P* = .002, *R*_s_ = 0.10; CDKN2A: *P* = 5.94E‐05, *R*_s_ = 0.13), but LIAS, LIPT1, and PDHB negatively correlated with TMB (LIAS: *P* = 9.45E‐18, *R*_s_ = ‐0.27; LIPT1: *P* = 1.17E‐06, *R*_s_ = ‐0.15; PDHB: *P* = 6.52E‐19, *R*_s_ = ‐0.28); CDKN2A expression was positively correlated with MSI (*P* = 7.56E‐07, *R*_s_ = 0.15), but PDHB and MTF1 negatively correlated with MSI (PDHB: *P* = 3.11E‐07, *R*_s_ = ‐0.16; MTF1: *P* = 7.95E‐05, *R*_s_ = ‐0.12) (Fig. S3, Supplemental Digital Content, http://links.lww.com/MD/N747).

## 4. Discussion

As a common regulatory mechanism in the organism, cell death is tightly linked to tumorigenesis and treatment.^[33]^ With the discovery of different cell death modalities, opportunities, and challenges have been brought to oncology research.^[34–37]^ Cuproptosis, as a novel cell death form, is of great significance to breast carcinoma research. This paper investigated and discussed the role of 10 cuproptosis-related genes in BRCA from multiple aspects and revealed their significance on breast cancer treatment and prognosis. This article is the first report to comprehensively analyze the association between cuproptosis-related genes and breast cancer to the best of our knowledge.

Cuproptosis-related genes were aberrantly expressed in various tumors by pan-cancer analysis of TIMER. Moreover, expression was generally downregulated than expression in the corresponding normal tissues; all cuproptosis-related genes except PDHB were abnormally expressed in BRCA. However, the UALCAN analysis found that all ten cuproptosis-related genes were abnormally expressed in BRCA; this different result may be due to the different algorithms used by TIMER and UALCAN. TIMER draws conclusions based on the Wilcox test, while UALCAN by the Student *t* test. In the UALCAN cohort, FDX1, LIAS, LIPT1, DLD, DLAT, PDHA1, MTF1, and GLS were lowly expressed in BRCA compared with normal breast tissue, but PDHB and CDKN2A were highly expressed. The results of TIMER and UALCAN are consistent except for PDHB. We used GEO data and immunohistochemistry to validate the expression analysis results from TCGA data of genes associated with cuproptosis in BRCA. We found that the performance of all genes except FDX1, DLAT, PDHB, GLS, and CDKN2A was consistent with the results of TCGA. The difference in sample size and population may contribute to this discrepancy. As cuproptosis-promoting genes, FDX1, LIAS, LIPT1, DLD, DLAT, and PDHA1 were under-expressed in breast cancer; at the same time, CDKN2A, which inhibited cuproptosis, was over-expressed in breast cancer, suggesting that cuproptosis might involve in the protective mechanism of BRCA.

Prognostic values of cuproptosis-related genes in BRCA were assessed based on mRNA sequencing and microarray data. By analyzing 2976 sequenced samples, we showed that higher expression of FDX1, LIAS, DLD, DLAT, MTF1, GLS, and CDKN2A was associated with longer OS. In evaluating survival before metastasis and recurrence, we found high FDX1, LIPT1, DLD, PDHB, MTF1, and GLS expression correlated with long RFS, and high expression of FDX1, LIAS, LIPT1, and PDHB was related to better DMFS. Therefore, FDX1, LIPT1, and PDHB have favorable prognostic values before breast cancer recurrence. Moreover, the hub gene FDX1 showed good performance in predicting OS, RFS, and DMFS; this also supported that cuproptosis might involve the protective mechanism of BRCA, and FDX1 might be a prognostic marker of BRCA.

After gene expression analysis based on clinicopathological parameters, we found almost no difference in cuproptosis-related gene expression among patients with different tumor stages. However, it is noteworthy that the cuproptosis-related gene expression values became lower with the progression of the tumor stage, suggesting that cuproptosis may inhibit breast cancer progression. Unlike individual tumor stages, cuproptosis-related genes were generally significantly differentially expressed in different subtypes of BRCA. A high mutation rate (51.3%) was found in cuproptosis-related genes in BRCA patients and OS, PFS, and DSS were poorer in patients with altered genes. The cuproptosis-related genes are mutation-prone in breast cancer, and mutations might result in poor prognoses. We identified the function of cuproptosis-related genes and the involved pathways related to the TCA cycle and protein lipoylation by enrichment analysis; this also echoes Peter Tsvetkov conclusion that cuproptosis is caused by copper binding to lipoylated proteins of the TCA cycle.^[7]^

Although breast cancer treatment has diversified, chemotherapy remains the cornerstone of breast cancer treatment.^[38,39]^ With the advent of the era of precision medicine, more and more researchers are focusing on the genetic and molecular mechanisms of disease treatment.^[40]^ A variety of cell death regulation modalities, like ferroptosis, pyroptosis, necroptosis, and autophagy, have been proven to affect the chemosensitivity of tumors.^[41–44]^ To further explore the value of cuproptosis in BRCA treatment, we evaluated the correlation of cuproptosis-related genes with the IC50 of doxorubicin, 5-fluorouracil, docetaxel, and cisplatin, which are commonly used chemotherapeutic drugs in breast cancer treatment.^[38,45,46]^ Genes associated with cuproptosis were generally negatively related to the IC50 of these 4 chemotherapeutic drugs, but there was no strong correlation. Although overexpression of cuproptosis-related genes might increase chemotherapy sensitivity, they had limited effect. Cuproptosis-related genes could be potential drug targets for researching and discovering new antitumor drugs.

Tumor tissue comprises tumor cells and other cells in the tumor microenvironment, such as fibroblasts, vascular endothelial cells, stromal cells, and immune cells.^[47,48]^ Among them, immune cell infiltration can markedly influence breast cancer patient’s therapeutic response and outcome.^[49,50]^ TIMER analysis found that cuproptosis-related genes were generally positively related to immunocyte infiltration, except for LIAS, with FDX1, DLAT, and GLS being more prominent. Moreover, high B cell, CD4+ T cell, CD8+ T cell, and dendritic cell infiltration improved cancer patients’ survival rate at 10 years. The above results complement the finding that highly expressed cuproptosis-related genes prolong breast cancer patients’ OS. For immune checkpoints, we showed that GLS was most strongly correlated with PDCDILG2. GLS and FDX1 were generally positively associated with immune checkpoint genes, while PDHB was generally commonly related to immune checkpoint genes. High TMB facilitates immunotherapy of multiple cancers.^[51]^ Although MSI is mostly mentioned in the immunotherapy of gastrointestinal tumors,^[52,53]^ studies of MSI evaluation of breast cancer immunotherapy have also been reported.^[54]^ LIAS, LIPT1, and PDHB were weakly negatively correlated with TMB, while DLAT, PDHA1, GLS, and CDKN2A was weakly positively related to TMB. PDHB and MTF1 were weakly negatively related to MSI, but CDKN2A was weakly positively related to MSI.

It has been proved that copper may promote tumorigenesis, progression,^[55,56]^ and angiogenesis,^[57,58]^ which is necessary for tumor progression and metastasis. Copper chelators have also been proposed as antitumor agents.^[59]^ The above view does not contradict our hypothesis that cell death due to copper may be a protective mechanism for breast cancer because cuproptosis is induced by copper ion carriers in an environment of copper accumulation. Analyzing the results of this paper, we proposed the conjecture that cuproptosis might participate in the protective mechanism of BRCA. Its key gene, FDX1, was prominent in BRCA prognoses and had the potential to become a drug target. Although generally associated with chemosensitivity, cuproptosis-related genes had a limited practical role. As for immune infiltration, cuproptosis-related genes were commonly correlated with various immunocyte infiltration levels in BRCA and affected prognoses. Thus, copper in the body is a double-edged sword. Future studies on cuproptosis should focus on the mechanisms involved in tumors and the development of novel antitumor drugs.

Our study indeed has limitations. First, we have only proposed the speculation that cuproptosis might associate with the protective mechanism of BRCA, which needs to be further explored by corresponding basic experiments. Second, we only used GEO data and immunohistochemistry data of HPA to verify gene expression levels in BRCA patients and did not perform in vivo or in vitro assays to validate them. Finally, this work is a retrospective study and requires prospective studies to corroborate each other.

In conclusion, this paper is a multidimensional exploration of the function of cuproptosis-related genes in BRCA patients. All cuproptosis-related genes were aberrantly expressed except PDHB in BRCA patients. The critical gene FDX1 might be a prognostic marker in breast cancer. Moreover, cuproptosis-related genes were related to immunocyte infiltration in breast cancer patients and impact prognoses. We proposed the hypothesis that cuproptosis may involve breast cancer’s protective mechanism, which needed to be verified by corresponding basic experiments. Future studies on cuproptosis should focus on the mechanisms involved in tumors and the development of novel antitumor drugs.

## Author contributions

**Conceptualization:** Jian Chen, Yingliang Li.

**Data curation:** Wei Cao.

**Formal analysis:** Jian Chen, Wei Cao.

**Funding acquisition:** Jia Zhu.

**Investigation:** Jian Chen, Jia Zhu.

**Methodology:** Jian Chen, Yingliang Li, Jia Zhu.

**Project administration:** Jian Chen, Yingliang Li, Jia Zhu.

**Resources:** Yingliang Li.

**Software:** Wei Cao.

**Supervision:** Jian Chen, Yingliang Li, Jia Zhu.

**Validation:** Jian Chen.

**Writing – original draft:** Jian Chen.

**Writing – review & editing:** Jia Zhu.

## Supplementary Material


