# Supplementary material for: Comprehensive analysis of the expression level, prognostic value, and immune infiltration of cuproptosis-related genes in human breast cancer
Source: Medicine (Baltimore). 2024 Oct 18;103(42):e40132. doi: 10.1097/MD.0000000000040132 (PMC11495725; doi:10.1097/MD.0000000000040132)
Supplement: Supplementary file 1 [file medi-103-e40132-s001.pdf]

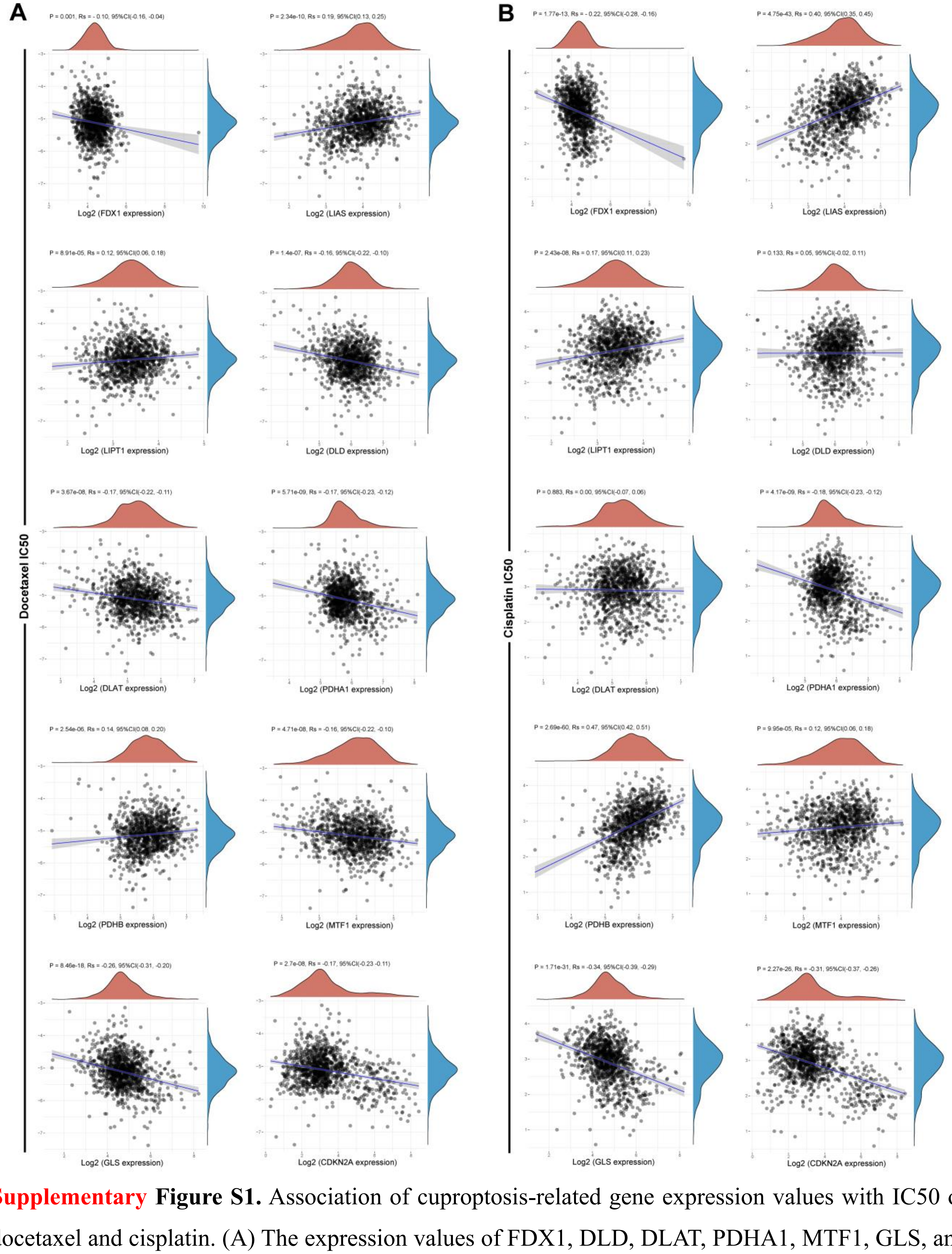

Supplementary **Figure S1.** Association of cuproptosis-related gene expression values with IC50 of docetaxel and cisplatin. (A) The expression values of FDX1, DLD, DLAT, PDHA1, MTF1, GLS, and CDKN2A were negatively related to IC50 of docetaxel, but LIAS, LIPT1, and PDHB were positively associated with IC50. (B) FDX1, PDHA1, GLS, and CDKN2A expression were negatively correlated

with IC50 of cisplatin, but the opposite outcomes were found in LIAS, LIPT1, PDHB, and MTF1. (IC50: disease-free survival)

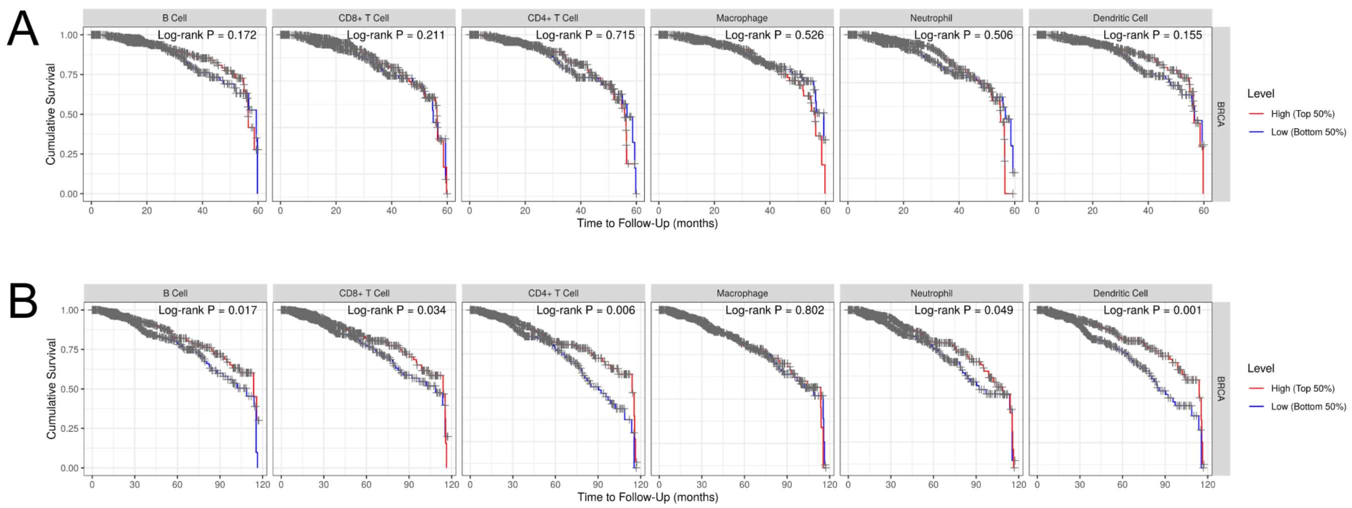

Supplementary **Figure S2.** The value of immunocyte infiltration on the prognosis of BRCA patients (TIMER). (A) Immunocyte infiltration was not related to cumulative survival at five years. (B) High infiltration of B cell, CD4+ T cell, CD8+ T cell, and Dendritic cell was related to favorable cumulative survival at ten years.

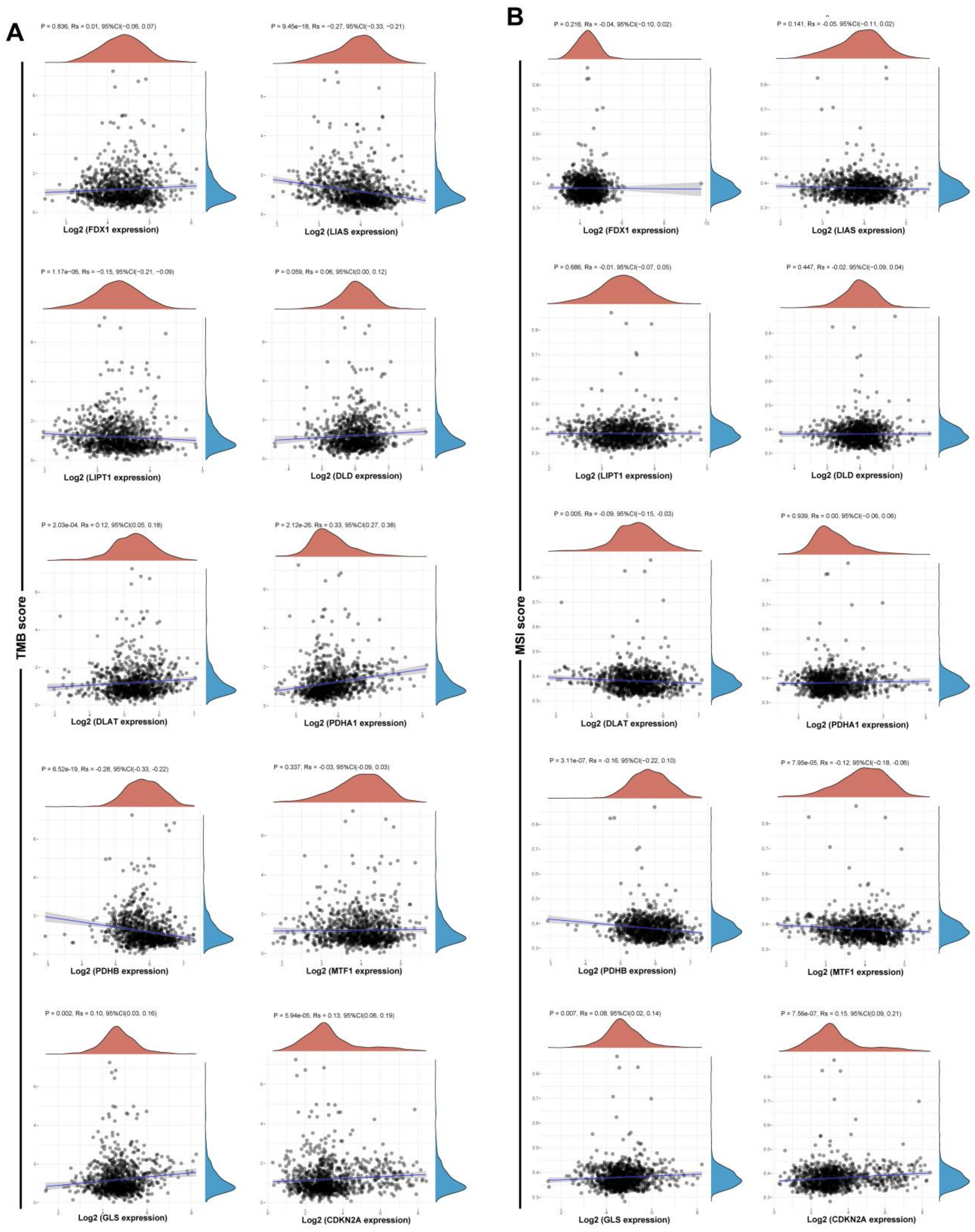

Supplementary Figure S3. Correlation of cuproptosis-related gene expression levels with TMB/MSI in BRCA. (A) DLAT, PDHA1, GLS, and CDKN2A expression was positively related to TMB, but LIAS, LIPT1, and PDHB were the opposite. (B) CDKN2A expression was positively related to MSI, but PDHB and MTF1 were the opposite. (TMB: tumor mutation burden, MSI: microsatellite instability)

Supplementary Table S1:

Results (P-value) of differential expression of cuproptosis-related genes in various tumors and normal tissues.

Tumor	FDX1	LIAS	LIPT1	DLD	DLAT	PDHA1	PDHB	MTF1	GLS	CDKN2A
BLCA	0.838008	0.150775	0.114673107	0.021329	0.52468	0.914431	0.243313	0.003651	0.19429779	0.003855
BRCA	3.78E-20	1.08E-30	2.86E-33	1.78E-10	0.002238	1.51E-11	0.122425	1.04E-09	1.29E-11	1.67E-31
CHOL	0.000135	1.02E-05	1.58E-08	0.003448	1.02E-05	0.000576	0.685809	2.26E-09	4.51E-09	4.33E-07
COAD	4.90E-07	0.018621	0.037629715	3.14E-07	0.007945	0.852605	0.004274	1.15E-11	0.000389497	6.48E-15
ESCA	0.052535	0.097271	0.001305762	0.097271	0.007098	0.599414	0.764371	0.150368	7.94E-05	0.079821
HNSC	0.073821	0.078855	0.524847786	0.510815	0.000116	0.589844	7.55E-08	0.131845	1.67E-16	0.000167
KICH	0.000609	0.841442	3.40E-11	0.002127	0.1105	0.485208	0.000437	1.24E-08	1.11E-10	1.78E-10
KIRC	2.63E-39	0.004002	0.006430388	7.85E-34	2.39E-29	1.46E-37	2.52E-38	3.25E-08	2.00E-25	1.69E-41
KIRP	1.71E-18	7.60E-05	0.000399353	0.064056	3.11E-08	0.006066	1.82E-11	6.19E-09	2.91E-05	1.23E-17
LIHC	0.050951	2.05E-11	2.28E-13	0.004824	6.38E-10	9.54E-10	8.25E-08	2.00E-07	6.88E-10	3.72E-25
LUAD.	2.44E-10	1.70E-07	0.146852591	0.336372	1.28E-10	4.87E-07	0.054308	0.000121	0.003925671	1.23E-12
LUSC	5.43E-09	1.67E-05	0.421893537	1.29E-10	3.30E-06	5.29E-14	1.95E-09	4.94E-05	1.68E-18	5.67E-08
PRAD	0.094747	0.00453	0.458677327	0.003104	0.015533	0.068872	0.289628	0.144669	0.023803138	1.93E-11
READ	0.007923	0.016124	0.703761349	0.000651	0.008385	0.230816	0.000376	0.013511	0.164534313	3.13E-05
STAD	3.03E-06	0.708736	0.000142831	0.003063	7.73E-08	8.91E-07	0.097048	0.109947	6.03E-09	7.85E-08
THCA	2.06E-21	8.68E-13	4.23E-17	2.90E-13	2.59E-07	6.91E-09	0.016636	9.15E-18	0.294623296	2.31E-24
UCEC	0.131344	3.86E-11	4.87E-14	0.246378	0.678005	0.009256	0.127661	6.02E-07	3.91E-11	4.77E-17

(BLCA: Bladder urotelial carcinoma, BRCA: Breast invasive carcinoma, CHOL: Cholangiocarcinoma, COAD: Colorectal adenocarcinoma, ESCA: Esophageal carcinoma, HNSC: Head and neck squamous cell carcinoma, KICH: Kidney Chromophobe carcinoma, KIRC: Kidney renal clear cell carcinoma, KIRP: Kidney renal papillary cell carcinoma, LIHC: Liver hepatocellular carcinoma, LUAD: Lung adenocarcinoma, LUSC: Lung squamous cell carcinoma ,PRAD: Prostate adenocarcinoma, READ: Rectal adenocarcinoma, STAD: Stomach adenocarcinoma, THCA: Thyroid carcinoma,UCEC: Uterine Corpus Endometrial Carcinoma)

Supplementary Table S2:
Results of the correlation between cuproptosis-related gene expression and immune cell infiltration in BRCA patients.

Gene	Purity		B Cell		CD8+ Cell		CD4+ Cell		Macrophage		Neutrophil		Dendritic Cell	
	P	Cor	P	Cor	P	Cor	P	Cor	P	Cor	P	Cor	P	Cor
FDX1	8.41E-12	-0.214	1.34E-10	0.204	2.95E-16	0.258	7.70E-04	0.108	1.40E-08	0.18	2.15E-15	0.253	1.62E-10	0.205
LIAS	2.28E-01	0.038	1.61E-04	-0.12	5.05E-02	0.063	1.69E-01	0.044	6.87E-03	0.086	2.44E-01	-0.038	1.01E-02	-0.083
LIPT1	5.22E-05	-0.128	5.46E-01	0.019	2.53E-16	0.258	2.53E-06	0.151	2.86E-05	0.133	8.39E-07	0.159	2.22E-01	0.04
DLD	3.35E-01	0.031	5.62E-06	0.145	3.33E-14	0.24	7.50E-03	0.086	1.56E-06	0.152	4.53E-09	0.189	5.73E-04	0.111
DLAT	2.25E-01	0.039	1.70E-11	0.213	1.41E-19	0.284	1.19E-03	0.104	7.54E-10	0.195	9.77E-17	0.265	1.92E-10	0.205
PDHA1	6.97E-05	0.126	1.89E-07	0.166	1.05E-02	0.082	6.46E-02	0.06	2.24E-02	-0.073	3.02E-04	0.117	4.16E-05	0.132
PDHB	5.08E-07	0.158	3.80E-01	-0.028	1.25E-01	0.049	9.84E-01	-0.001	3.00E-05	0.133	4.75E-01	-0.023	8.60E-02	-0.056
MTF1	1.68E-01	-0.044	5.97E-03	0.088	1.63E-25	0.325	2.11E-12	0.224	1.17E-17	0.268	2.13E-24	0.322	1.25E-11	0.217
GLS	1.18E-09	-0.191	5.57E-07	0.159	1.11E-35	0.384	3.63E-16	0.259	8.51E-20	0.285	2.05E-41	0.418	4.08E-31	0.364
CDKN2A	1E-01	-0.052	2.88E-06	0.149	2.45E-01	-0.037	7.00E-04	0.109	6.67E-03	-0.086	8.83E-04	0.108	3.61E-05	0.134
